# The Way to Pursue Truly High-Performance Perovskite Solar Cells

**DOI:** 10.3390/nano9091269

**Published:** 2019-09-05

**Authors:** Jia-Ren Wu, Diksha Thakur, Shou-En Chiang, Anjali Chandel, Jyh-Shyang Wang, Kuan-Cheng Chiu, Sheng Hsiung Chang

**Affiliations:** 1 Department of Physics, Chung Yuan Christian University, Taoyuan 32023, Taiwan; 2 Center for Nano Technology, Chung Yuan Christian University, Taoyuan 32023, Taiwan

**Keywords:** perovskite solar cells, hot-carrier characteristics, quantized electron transport layer, quantized hole transport layer

## Abstract

The power conversion efficiency (PCE) of single-junction solar cells was theoretically predicted to be limited by the Shockley–Queisser limit due to the intrinsic potential loss of the photo-excited electrons in the light absorbing materials. Up to now, the optimized GaAs solar cell has the highest PCE of 29.1%, which is close to the theoretical limit of ~33%. To pursue the perfect photovoltaic performance, it is necessary to extend the lifetimes of the photo-excited carriers (hot electrons and hot holes) and to collect the hot carriers without potential loss. Thanks to the long-lived hot carriers in perovskite crystal materials, it is possible to completely convert the photon energy to electrical power when the hot electrons and hot holes can freely transport in the quantized energy levels of the electron transport layer and hole transport layer, respectively. In order to achieve the ideal PCE, the interactions between photo-excited carriers and phonons in perovskite solar cells has to be completely understood.

## 1. Introduction

The bounded electrons of inorganic and organic semiconductors can be efficiently and instantaneously excited from the ground state to the excited state when the photon energy of the incident lightwaves is higher than the absorption bandgap. However, the photo-excited electrons (hot electrons) in the light-absorbing materials (LAMs) have to relax to the meta-stable state (conduction band minimum (CBM) or lowest unoccupied molecular orbital (LUMO)) due to the ultrafast thermallization process [1,2,3,4], which results in the intrinsic potential loss and thereby limits the power conversion efficiency (PCE) of single-junction solar cells to be a moderate value of 33.7% [5]. The physical concept of the Shockley–Queisser (S–Q) limit can be understood as the following descriptions. When then LAM has a large bandgap, the broadband sun light cannot be efficiently absorbed by the wide-bandgap material. Therefore, the photocurrent density of solar cells can be increased with a decrease in the absorption bandgap of the active layer. For example, the photocurrent density of single-crystalline Si solar cells (~42 mA/cm^2^) is always higher than that of single-crystalline GaAs solar cells (~29 mA/cm^2^) because the absorption bandgap of crystalline Si (1.1 eV) is lower than that of crystalline GaAs (1.43 eV). When the active layer is a wide-bandgap material, the photo-excited electrons have to relax to the meta-stable state, which indicates that the highest potential difference between the cathode electrode and the anode electrode is equal to *E_g_*/*e*. *E_g_*and *e* are the absorption bandgap of the active layer and the electric charge, respectively. Usually, the potential difference between the cathode electrode and the anode electrode equals to the open-circuit voltage (V_OC_) which is defined by the current density–voltage (J–V) curve of solar cells. As we know that the V_OC_ of a solar cell is proportional to the absorption bandgap of the active layer. For example, the V_OC_ of single-crystalline Si solar cells (~0.738 V) is lower than that of single-crystalline GaAs solar cells (~1.127 V). According to the S–Q limit, it is impossible to simultaneously obtain the high V_OC_ and the high photocurrent density (short-circuit current density, J_SC_), which results in an optimal absorption bandgap of 1.34 eV for the highest PCE of 33.7%.

In the past several decades, physical and chemical scientists were trying to achieve the highest PCE of 33.7% by using the different types of solar cells. When the exciton binding energy of LAMs is lower than the thermal energy, the planar thin-film structure can be used to construct the high-performance solar cells, such as the crystalline Si [6,7], crystalline GaAs [8,9] and crystalline InP [10,11] solar cells. When the exciton binding energy of the LAMs is higher than the thermal energy, the P:N nanocomposite thin-film structures have to be used to increase the photovoltaic performance, such as the organic bulk-heterojunction solar cells [12,13,14] and dye-sensitized solar cells (DSSCs) [15,16,17]. Although the PCE of organic photovoltaics (OPVs) and DSSCs is significantly lower than that of the planar thin-film inorganic semiconductor-based solar cells, the cost-effective OPVs and DSSCs still received a lot of attentions in the past two decades. Thanks to the fundamental investigations on the OPVs and DSSCs, the PCE of perovskite solar cells has dramatically increased from 3.8% [18] to 25.2% [19] by using the solution-processed methods.

It is amazing that the high-efficiency perovskite solar cells can be realized by using the low-temperature solution-processed methods because the presence of high-density defects in the active layer [20,21,22] usually can simultaneously reduce the V_OC_, J_SC_ and fill factor (FF) of solar cells. It is well known that high-efficiency perovskite solar cells can be explained mainly due to the large absorption coefficient [23,24], moderate refractive index [25,26], low exciton binding energy [27,28], long exciton (carrier) lifetime [29,30] and long exciton (carrier) diffusion length [31,32]. In addition, the high PCE of perovskite solar cells also relieson the efficient energy transfer at the perovskite/electron transport layer (ETL) and perovskite/hole transport layer (HTL) interfaces. The highest PCE of perovskite solar cells is theoretically predicted to be an attractive value of 31% [33], which is also limited by the prediction from the S–Q limit.

To pursue truly high-performance solar cells, it is necessary to reduce the intrinsic potential loss via increasing the hot-carrier lifetimes of LAMs. The hot-electron lifetimes of GaAs, Si and InP crystals are 1.5 ps [34], 0.18 ps [35] and 3.4 ns [36], respectively. In general, the lower phonon energy corresponds to the longer hot-electron lifetime [37,38]. The ultrashort hot-electron lifetimes mean that the photo-excited electrons must relax to the meta-stable state to form excitons. In recent reports, the lifetime for the hot electrons in perovskite crystals has been related to the +1 cation [39]. Furthermore, the hot-electron lifetime (diffusion length) of MAPbI_3_ thin films was determined to be longer than 20 ps (600 nm) by using transient absorbance spectroscopy [40]. The long-lived hot-carrier mediated light emission was also observed in formamidinium tin triiodide perovskites [41]. The existence of long-lived hot electrons means that it is possible to realize truly high-performance solar cells when crystalline perovskite thin films are used as the LAM.

In this review, we discuss the hot-carrier characteristics and the ways for hot-carrier extractions in the energy–space diagrams. A theoretical point of view is proposed in order to understand how the single-junction hot-carrier solar cells can be realized. Finally, the practical issues are discussed in order to assess the possibility for the realization of perovskite-based hot-carrier solar cells.

## 2. Light-Materials’ Interactions: Excited Bounded Electrons

**Figure 1:**
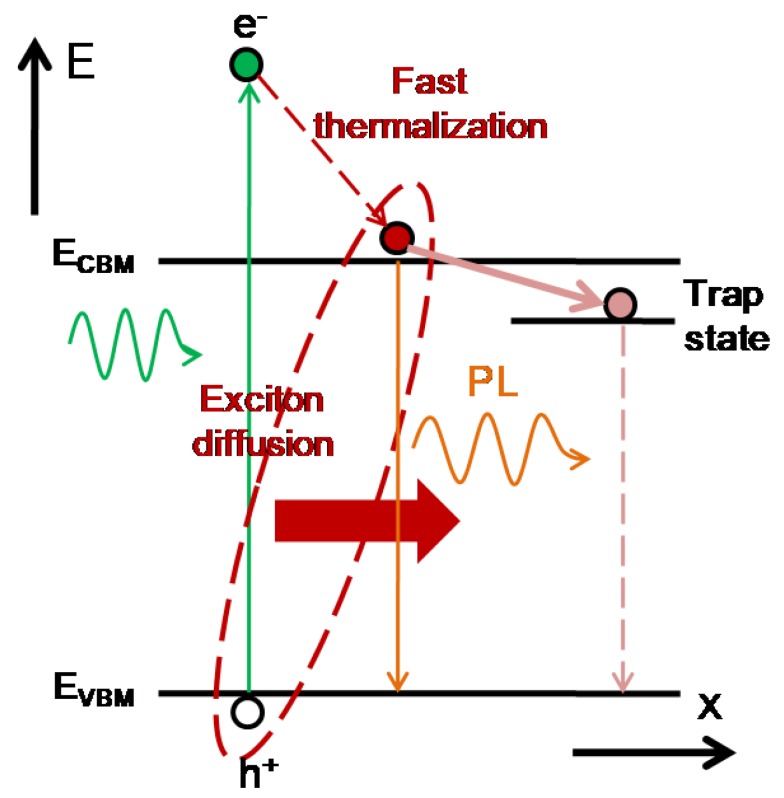
Photo-excited carrier dynamics in an energy–space diagram.

Photon energy can be efficiently converted to electrical power by using p-type materials due to the high absorption coefficient. Figure 1 shows the carrier dynamics of photo-excited electrons in an energy–space diagram. When the incident photons are absorbed by a p-type material, the electrons in the ground state can transit to the excited state to form hot carriers. The hot carriers can be viewed as the oscillating charged particles, which can coherently and incoherently collide with the lattice vibrations (photons). The coherent collisions between the hot carriers and phonons can result in Raman scattering emissions. The incoherent collisions between the hot carriers and photons can result in the photoluminescence (PL) emissions. In addition, the hot-carrier mediated PL emissions can be observed in the perovskite thin film due to the slow thermalization process [41].

In general, the hot-carrier lifetime of organic LAMs is shorter than 100 fs. Therefore, it is not easy to observe the hot-carrier dynamics of organic materials by using the femtosecond time-resolved photoluminescence (FTR-PL) technique due to the limited instrument response function (IRF ~150 fs) [42,43]. The ultrashort hot-carrier lifetime is mainly due to the large optical phonon energy of organic materials [44], which results in an extremely short hot-carrier diffusion length. It means that the hot carriers rapidly decay to the meta-stable state in organic materials via the thermalization (downhill relaxation) process [2,45]. Then, the electrons in the lowest unoccupied molecular orbital (LUMO) and the holes in the highest occupied molecular orbital (HOMO) are mutually attracted to form excitons. In conjugated small organic molecules, the exciton binding energy can be reduced by increasing the conjugation due to the delocalization effect [46]. In general, the exciton-binding energy of organic LAMs can be a wide range from 0.3 eV to 1.0 eV [47,48], which depends on the degree of delocalization of electron-hole pairs. This means that the larger exciton binding energy corresponds to the shorter (smaller) exciton radius (volume). From the concept of allowable excitation density, the smaller exciton volume results in the higher exciton generation rate (absorption coefficient). Due to the high density of excitons, the exciton diffusion length and exciton lifetime of organic materials can be lower than 1 nm and 1 ns, respectively.

**Figure 2:**
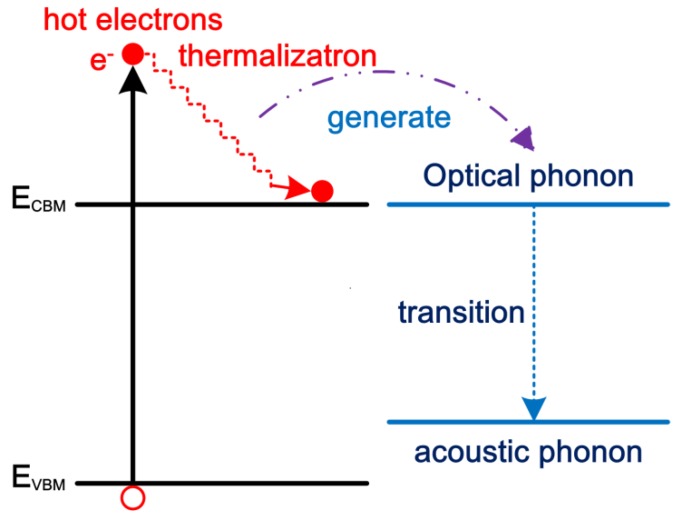
Hot carrier-optical phonon energy transfer and thermalization process.

It can be predicted that the hot-carrier lifetime of inorganic materials [34,35,36] is longer than that of organic materials because the optical phonon energy of inorganic materials [49,50] is lower. The optical phonon energy and hot-carrier lifetime of various materials are listed in Table 1 [34,35,36,37,38,49,50,51,52,53]. During the hot-carrier thermalization process in a polar semiconductor as shown in Figure 2, the energy of the hot carriers has to be firstly transferred to the longitudinal optical (LO) phonons. Then, the transition from the LO phonons to acoustic phonons results in the lattice heating. With the propagation of acoustic phonons, the thermal energy can transfer to the surroundings. This means that there are three ways that can be used to slow down the hot-carrier thermalization process. The energy transfer rate from hot carriers to LO phonons is intrinsic fast in non-polar materials [54,55], such as Si and Ge. In a polar CH_3_NH_3_PbI_3_ (MAPbI_3_) crystal thin film, the energy transfer rate from hot carriers to LO phonons can be delayed due to the formation of hot polarons [55], which results in a long hot-carrier cooling time. As we know that hot polarons are quasiparticles, which describes the interaction between the hot carriers and polar lattices. Therefore, the formation of hot polarons can delay the transfer rate from hot carriers to LO phonons. This was firstly explained due to a phonon bottleneck effect [56]. In addition, the propagation of acoustic phonons in perovskite crystals is theoretically predicted to be slow due to the use the insulating organic cation [57], which also can generate the up-conversion of acoustic phonons to re-heat the hot carriers and thereby increases the hot-carrier lifetime [53]. Up to now, the methods to delay the energy transition from LO phonons to acoustic phonons have not yet been proposed for increasing the hot-carrier lifetime in polar perovskite crystals.

## 3. Hot-Carrier Extraction at a Light-Absorbing Material/Electron Transport Layer (LAM/ETL) Interface

**Figure 3:**
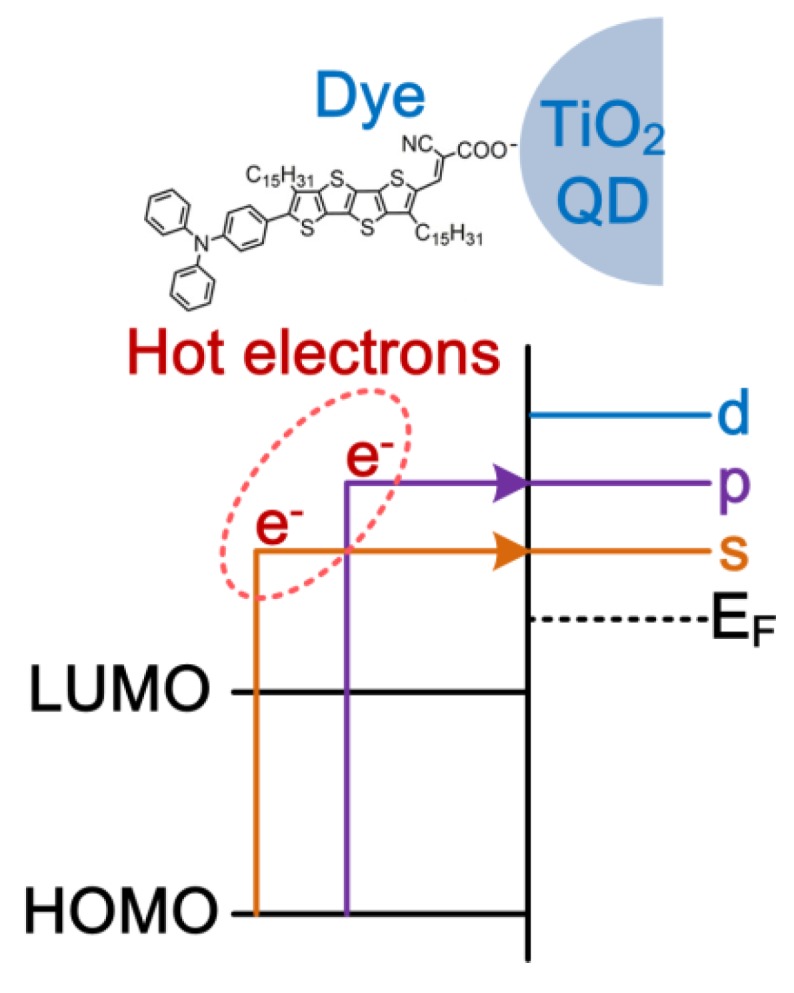
Hot-electron injection from dyes to the quantized energy levels of TiO_2_ quantum dots (QDs).

The hot-electron injection from organic fused thiophene-based dyes to TiO_2_ nanoparticles (NPs) was investigated for the first time by using a FTR-PL technique [58], which was used to explain the abnormal high V_OC_ of 0.93 V in DSSCs with an iodide/triliodide based electrolyte [50]. The dyes can be adsorbed on the surface of TiO_2_ NPs due to the electrical attraction between the anchoring group of dyes and the oxygen defect of TiO_2_ NPs. Therefore, it can be understood that the high V_OC_ is due to the hot-electron injection from the dyes to the higher quantized energy levels of the TiO_2_ NPs, as shown in Figure 3. In this study, the average diameter of the TiO_2_ NPs is about 10 nm [59], which is about 3 times of the exciton radius. Therefore, it can be predicted that the quantized energy levels can be created in the TiO_2_ quantum dots (QDs) [60].

The efficient hot-electron extraction in OPVs has not yet been reported in literature, which is probably due to the fact that the diffusion process is needed for the hot carriers to reach the region of charge transfer radius [61,62]. For example, the hot electrons in poly(3-hexylthiophene-2,5,-diyl) (P3HT) polymers is rapidly decayed from the excited state to the LUMO energy level due to the ultrafast self-localization process (~100 fs) [2,43], which suppresses the hot-electron diffusion. This means that it is possible to realize organic hot-carrier photovoltaics when the ultrafast self-localization process can be reduced by increasing the delocalization quantum-assisted transport within long polymer chains [46].

The efficient hot-electron extraction in perovskite solar cells has not yet been discussed in literature. However, the abnormal high V_OC_ (=1.61 V) of inverted-type MAPbBr_3_ based solar cells is probably due to the efficient hot-electron extraction from the MAPbBr_3_ nano-crystals to the indene-C_60_-bisadduct (ICBA) ETL [63]. In this study, the poly(3,4-ethylenedioxythiophene):polystyrene sulfonate (PEDOT:PSS (1:6 wt%)) thin film and ICBA thin film are used as the HTL and ETL, respectively. The Fermi level of PEDOT:PSS thin film and the LUMO energy level of ICBA thin film are −5.1 eV [64,65] and −3.9 eV [66,67], respectively, which results in a S.-Q limited V_OC_ of 1.2 eV. The high V_OC_ of MAPbBr_3_ based solar cells means that the hot electrons have to be extracted by the LUMO+1 and/or LUMO+2 of the ICBA thin film because the PEDOT:PSS thin film is a metal-like conductive polymer [68]. The photovoltaic performances of high-V_OC_ perovskite based solar cells are listed in Table 2 [63,69,70]. The three types of perovskite solar cells both contain bromide elements in the active layer, which suggests that the bromide-based perovskite thin films probably have longer hot-carrier lifetimes. In addition, the long hot-carrier lifetime and diffusion length were observed in a MAPbI_3_ perovskite thin film by using transient absorbance spectroscopy [40], which means that the realization of high-performance hot-carrier perovskite solar cells is possible.

## 4. Hot-Carrier Extraction at a LAM/Hole Transport Layer (HTL) Interface

**Figure 4:**
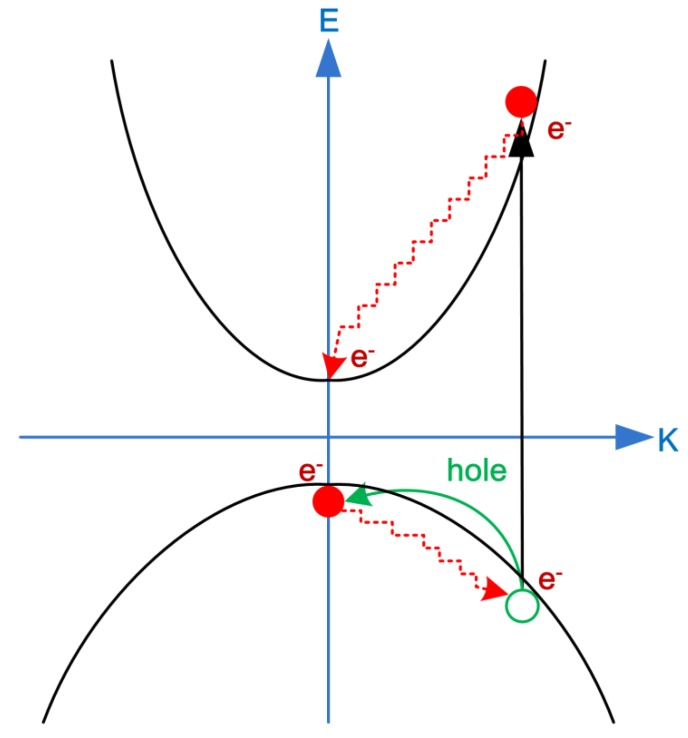
Energy diagram of hot-hole and hot-electron relaxations.

As we know that the bounded electrons are not excited from the valence band maximum (E_VBM_) when the photon energy of the incident lightwaves is higher than the absorption bandgap of materials. Therefore, the hot-hole relaxation process has to be considered in order to realize the hot-carrier solar cells. Figure 4 shows the hot-hole and hot-electron dynamics in the energy diagram. For example, the hot-hole and hot-electron relaxation times of a MAPbI_3_ thin film are 100–500 fs and 1–5 ps [71], respectively, which means that it is more difficult to collect the hot holes in perovskite thin films by using a hot-hole selective layer due to the sub-picosecond relaxation time. Fortunately, there is experimental evidence of transient absorbance spectra to show that the hot holes in a CsPbI_3_ thin film can be efficiently extracted by the capping layer of P3HT thin film within a few 100 fs [72]. However, the hot-hole extraction process from the perovskite thin film to the P3HT thin film is not yet completely understood. Further experiments are needed to demonstrate that the hot-hole extraction can increase the V_OC_ of perovskite solar cells.

The extraction efficiency of hot holes is also related to the carrier diffusion coefficient of perovskite thin films because the hot holes have to diffuse into the region of the charge transfer radius at the perovskite/HTL interface. According to the Einstein relation ($D=\mu k_{B}T/e$), the carrier diffusion coefficient (*D*) is proportional to the carrier mobility (*μ*), where *k_B_T* is the thermal energy and *e* is the electric charge. The carrier drift equation ($\mu =e\tau /m^{*}$) shows that the carrier mobility is proportional (inversely proportional) to the carrier relaxation time (effective mass), where *τ*and m^*^are the carrier relaxation time and carrier effective mass, respectively. Therefore, the smaller hot-hole effective mass corresponds to the longer hot-hole diffusion length and thereby results in the higher extraction efficiency of hot holes. Interestingly, the hole mobility can be higher than the electron mobility when the LAM is CsPbBr_3_ or CsPbCl_3_, which is due to the lower hole effective mass [73]. However, the propagation characteristics of hot holes are not yet completely understood.

## 5. Theoretical Point of View

**Figure 5:**
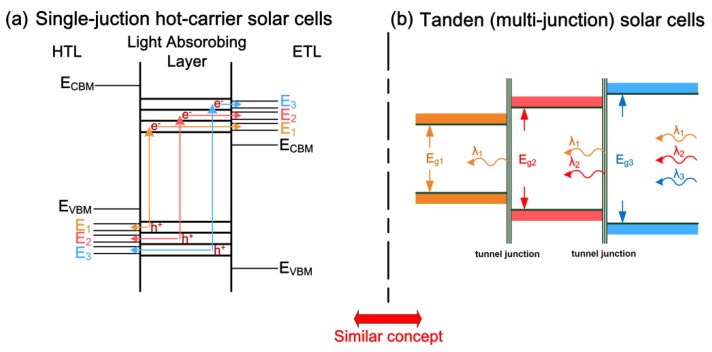
Energy diagrams. (**a**) Single-junction hot-carrier solar cell. (**b**) Tandem solar cell.

The highest PCE of single-junction hot-carrier solar cells was theoretically predicted to be 66% under one sun illumination [74]. Figure 5a shows the energy diagram of a single-junction hot-carrier solar cell. In the single-junction solar cell, the high-energy and low-energy incident photons are absorbed by the electrons in the deeper levels and in the shallower levels of the valence band, respectively. When the electrons in the valence band are excited to the conduction band, the hot electrons in the higher energy levels and in the lower energy levels both have to be directly extracted in order to avoid ultrafast potential loss. As to the hot holes, they also have to be directly extracted in order to keep the original potential.

The concept of single-junction hot-carrier solar cells is similar to the tandem (multi-junction) solar cells which has a highly theoretical PCE of 68% under one sun illumination [75]. Up to now, the highest PCE of tandem solar cells is 39.2% under one sun illumination, which is significantly higher than the highest PCE (29.1%) of single-junction GaAs solar cells [19]. When the different absorption ranges in a single LAM are viewed as the individual materials with the different absorption bandgaps, the energy diagram of a tandem solar cell can be plotted in Figure 5b. The PCE of tandem solar cells is strongly related to the performance of the tunnel junctions [76] with an ultrafast carrier dynamic [77]. In single-junction hot-carrier solar cells, the double-barrier resonant tunneling structure was proposed as the energy selective contact of hot carrier solar cells [78] due to the sub-picosecond carrier extraction ability [79]. Therefore, it can be believed that the highly efficient single-junction hot-carrier solar cells can be realized when the ultrafast carrier lifetimes of hot carriers in the LAM can be increased from the sub-picosecond time scale to sub-nanosecond time scale.

In order to efficiently collect the hot carriers at the different energy levels, a multi-band ETL and a multi-band HTL have to be used to extract the dispersive hot carriers under a broadband excitation. T 5ashows the energy diagram of an ideal hot-carrier solar cell. The E_CBM_ of HTL (E_VBM_ of ETL) has to be higher (lower) than the E_3_ in the conduction band of ETL (E_3_ in the valence band of HTL) in order to block the hot electrons (hot holes), which can help the collection of hot carriers. If the high-energy hot carriers can be efficiently collected by the E_3_, the hot electrons (hot holes) have to freely transport within the E_3_ of ETL (HTL) without the energy transitions from the E_3_ to the E_2_ and/or E_1_. Fortunately, the photo-excited carriers can be collected and transport in the quantized energy levels of Si-doped quantum dots, which increases the V_OC_ from 0.78 V to 0.91 V [80]. Therefore, it can be expected that the hot electrons and hot holes can freely transport in the quantized energy levels of ETL and HTL, respectively. In addition, n-type graphene quantum dots [81,82,83] and p-type graphene quantum dots [84,85,86] might have the potential as the multi-band ETL and multi-band HTL of hot-carrier solar cells, respectively.

In addition, a theoretical approach is used to calculate the J–V curves of single-junction solar cells when the selective contacts are used to collect the hot carriers [87]. Their simulation results show that the *e*×V_OC_ of hot-carrier solar cells can larger than the absorption bandgap of LAMs. For example, *e*×V_OC_ of the GaAs solar cell is 1.85 eV which is larger than the absorption bandgap of crystalline GaAs.

## 6. Experimental Challenges and Opportunities

Up to now, the LAMs of highly efficient perovskite solar cells were fabricated by using solution-processed methods [26,28,88,89,90,91], which means that the defect density of LAMs remains high. Although the shallow defects of perovskite thin films do not significantly influence the photovoltaic performance [92,93], the formation of defects can increase the exciton binding energy and optical phonon energy of perovskite thin films [51,94]. Therefore, the defect-mediated phononic properties of perovskite thin films have to be investigated in order to understand how to realize hot-carrier solar cells.

The grain size of solution-processed perovskite thin films can be increased from several hundred nanometers to several micrometers by adding the small molecules [95] or with the solvent annealing process [96]. The average crystal domain size of perovskite thin films is smaller than averaged grain size, which indicates that the perovskite thin films are composed of multi-crystalline grains [97]. It means that the residual stress in a multi-crystalline perovskite thin film [98] can also influence the phononic properties, which might dominate the hot-carrier characteristics. In other words, the defect-mediated phononic properties [99] and/or crystal distortion-mediated phononic properties [100] have to be considered when the perovskite thin films are fabricated on top of amorphous substrates or poly-crystalline substrates by using solution processes or thermal evaporation methods. However, the preferred oriented perovskite thin films can be fabricated on top of the single-crystalline substrates by using the spin-coating method [101], which was observed by measuring the two-dimensional X-ray diffraction patterns.

Conceptually, the existences of defects and lattice distortions should decrease the hot-carrier lifetimes in the LAMs, which are predicted to impede the development of hot-carrier solar cells. Therefore, the development of single-crystalline perovskite bulks plays an important step for the realization of high-performance optoelectronic devices. Two years ago, the single-crystalline perovskites were grown on top of various substrates, such as indium tin oxide (ITO), quartz and silicon wafer, which were used as the X-ray detector [102]. The strategy is to modify the surface of the substrates with a NH_3_-Br-teminated self-assembling molecules monolayer, which provides a seeding layer to grow the single-crystalline MAPbBr_3_. The long carrier lifetime of the single-crystalline MAPbBr_3_ is 692 ns, which indicates the low defect density. Therefore, the hot-carrier lifetimes of single-crystalline perovskites can be expected to be longer than that of poly-crystalline perovskite thin films.

When the substrate (Au/p^+^-type wafer) is the anode side, the single-crystalline perovskite has to be grown on top of a quantized HTL. Then, a quantized ETL has to be fabricated on top of the single-crystalline perovskite. A transparent conductive oxide has to be deposited on top of the device as the cathode electrode. Therefore, it can be imagined that the device architecture is Au/p^+^-type wafer/quantized HTL/single-crystalline perovskite/quantized ETL/transparent conductive cathode. The p^+^-type wafer has to be a large-bandgap material in order to block the hot electrons from the single-crystalline perovskite. The quantized HTL and the quantized ETL can be a p-type quantum well (QW) structure [103] and a double-barrier resonant-tunneling structure [78], respectively. In addition, the transparent conductive cathode is used to block the hot holes from the single-crystalline perovskite. The potential candidates as the p^+^-type substrate, HTL, ETL and transparent conductive anode are listed in Table 3. The Au coated p^+^-type GaN, AlN or SiC wafer can be used as the anode electrode. The epitaxial growth process of GaN/AlN QW [104] or AlGaN QDs [105] on top of the GaN or AlN wafer is a mature technique by using metal organic chemical-vapor deposition (MOCVD) or molecular beam epitaxy (MBE). However, the barrier high and physical size of the QW or QDs has to be investigated and designed in order to be used as the quantized HTL for the collection of hot holes. P-type graphene QDs can be produced by using microwave-assisted heating method [106] or pulsed laser ablation method [107]. And, the p-type graphene QDs thin film can be deposited on top of the p^+^-type wafer by using the spin-coating method. Although, the single-crystalline MAPbBr_3_ has been demonstrated that can be grown on various substrates with a surface modification method [102]. The contact at the MAPbI_3_/HTL interface, which should strongly influence the collection efficiency of hot holes, has not yet investigated. It is predicted that the [6,6]-phenyl-C_61_-butyric acid methyl ester (PCBM)/bathocuproine (BCP) QW, ZnO QDs [108] or TiO_2_ QDs [109] can be used to collect the hot electrons from the single-crystalline perovskite. PCBM molecules can be dissolved in low-polarity solvents, such as chlorobenzene and toluene, which can be directly spin-coated on top of MAPbI_3_ thin films. BCP molecules, ZnO QDs and TiO_2_ QDs are usually dissolved in isopropanol (IPA) which is a polar solvent. Therefore, the BCP, ZnO QDs or TiO_2_ QDs thin film cannot be directly spin-coated on top of MAPbI_3_ thin films. The contact at the hydrophobic ETL/hydrophilic MAPbI_3_ interface can be improved by slightly roughening the surface of MAPbI_3_ thin film [91]. Then, the Al-doped ZnO, Ga-doped ZnO or Al-Ga co-doped ZnO thin film can be used as the transparent conductive cathode because of the metal-like electrical conductivity which can directly collect the hot electrons without the additional potential loss. However, the high-quality transparent conductive cathodes are usually deposited by using the radio-frequency magnetron sputtering method, which can damage the MAPbI_3_ thin film due to the excessive energy bombardment during the deposition process [110]. To resist the excessive energy bombardment, an inorganic thin film has to be used as the buffer layer in between the MAPbI_3_ thin film and transparent conductive cathode [110].

The realization of hot-carrier solar cells has to rely on the long-lived hot carriers in the active layer and the efficient collections of hot carriers by the HTL and ETL. The hot-carrier lifetime and hot-carrier diffusion length of crystalline perovskite thin films can be longer than 100 ps and 600 nm, respectively, which indicates that the hot carriers are possibly collected without potential loss. However, the hot carriers have to be rapidly collected before the ultrafast thermalization process, which means that the collection times of hot carriers have to be faster than the hot-carrier lifetimes. For example, the hot-carrier lifetime of the LAM and the hot-carrier collection time of a double-barrier resonant tunneling structure can be 100 ps and 0.4ps, respectively. Furthermore, the hot-carrier collection efficiency can be calculated by h = 1/t_1_/(1/t_1_ + 1/t_2_) [58], where t_1_ and t_2_ are hot-carrier collection time and hot-carrier lifetime, respectively. Therefore, the calculated hot-carrier collection efficiency equals to 99.6%, which means that it is worthwhile to develop a double-barrier resonant tunneling structure as the selective contact of hot-carrier perovskite solar cells. In this review, we suggest that the quantized HTL and quantized ETL can be used to collect the dispersive hot holes and hot electrons, respectively. The collection times of hot carriers will dominate the collection efficiency. Therefore, the barrier height and physical size of the quantized HTL and quantized ETL has to be varied in order to decrease the collection times of hot carriers. Conceptually, the collection times of hot carriers are also influenced by the crystallinity (carrier mobility) of the HTL and ETL. Therefore, the formation of high-quality quantized HTL, quantized ETL and perovskite thin film is necessary in order to realize hot-carrier solar cells.

## 7. Conclusions

Recent progress in the understanding of the light-perovskite interactions shows that the realization of highly efficient hot-carrier solar cells is possible because the long-lived hot polarons can be formed and thereby delays the thermalization process. Conceptually, the extraction of hot electrons and hot holes can increase the open-circuit voltage (V_OC_). The hot-electron injection was firstly observed in the dye-sensitized solar cells, which significantly increased the V_OC_ from 0.75 V to 0.93 V. The long-lived hot electrons in the organic fused thiophene-based dyes were observed by using the femtosecond time-resolved photoluminescence technique.

The highest V_OC_ of CH_3_NH_3_PbBr_3_ solar cells is 1.61 V, which is far larger than the potential difference (1.2 V) between the LUMO energy level of the electron transport layer (ICBA thin film) and the Fermi level of the hole transport layer (PEDOT:PSS thin film). The abnormal high V_OC_ of perovskite solar cells can be explained due to the efficient collection of hot electrons by the LUMO+1 and/or LUMO+2 of the ICBA thin film. In addition, the P3HT polymer thin film was used as the hot-hole selective contact layer, which was observed by using the femtosecond transient absorbance spectra.

To realize truly high-performance hot-carrier perovskite solar cells, it is necessary to simultaneously collect the hot electrons and hot holes without the additional potential loss. We have proposed a device architecture which might be used to achieve the desired power conversion efficiency. The device architecture is Au/p^+^-type GaN wafer/quantized hole transport layer (QHTL)/crystalline perovskite/quantized electron transport layer (QETL)/transparent conductive cathode. Ideally, the hot holes and hot electrons in the crystalline perovskite have to be collected by the QHTL and QETL, respectively. Therefore, it is necessaryto investigate the ultrafast energy transfer dynamics of hot carriers from the crystalline perovskite to the QHTL and QETL by varying the barrier high and physical size of the quantum wells or quantum dots.

## Figures and Tables

**Table 1:** Optical phonon energies (*E*_Phonon_) and hot-carrier lifetimes (τ_hc_) of various inorganic materials and organic materials. TPA-TTAR-A: triphenylamine-tetrathienoacene-acceptor.

Materials	GaAs	Si	InP	P3HT	TPA-TTAR-A	CH_3_NH_3_PbI_3_	HC(NH_2_)_2_PbI_3_
*E*_Phonon_ (meV)	40	60	42	None	None	25	11.5
τ_hc_ (ps)	1.5	0.18	3400	<0.1	1.01	20	124
Ref.	[34]	[35,36]	[37,38]	[49]	[50]	[40,51]	[52,53]

**Table 2:** Photovoltaic performance of bromide-based perovskite solar cells.

Active Layer	MAPbBr_3_	CsPbI_2_Br	CsPb_0.97_Tb_0.03_Br_3_
ETL/LUMO	ICBA/−3.9 eV	TiO_2_/−4.1 eV	TiO_2_/−4.1 eV
HTL/E_F_ or HOMO	PEDOT:PSS/−5.1 eV	Spiro-OMe TAD/−5.2 eV	NiO_x_/−5.1 eV
V_OC_ (V)	1.61	~1.3	1.57
J_SC_ (mA/cm^2^)	6.04	~12	8.21
FF (%)	77.0	~74	79.6
Ref.	[63]	[69]	[70]

**Table 3:** Potential candidates as the p^+^-type substrate, hole transport layer (HTL), electron transport layer (ETL) and transparent conductive anode.

p^+^-Type Substrate	HTL	ETL	Transparent Conductive Cathode
GaN	GaN/AlGaN QW	PCBM/BCP QW	Al-doped ZnO
AlN	AlGaN QDs	ZnO QDs	Ga-doped ZnO
SiC	p-type graphene QDs	TiO_2_ QDs	Al-Ga co-doped ZnO
